# Size matters! Association between journal size and longitudinal variability of the Journal Impact Factor

**DOI:** 10.1371/journal.pone.0225360

**Published:** 2019-11-22

**Authors:** Dorothea Koelblinger, Georg Zimmermann, Silke B. Weineck, Tobias Kiesslich

**Affiliations:** 1 Research Office, Paracelsus Medical University, Salzburg, Austria; 2 Department of Neurology, Spinal Cord Injury and Tissue Regeneration Center Salzburg, Paracelsus Medical University, Salzburg, Austria; 3 Department of Neurology, Christian Doppler Medical Centre, Paracelsus Medical University, Salzburg, Austria; 4 Department of Mathematics, Paris-Lodron University of Salzburg, Salzburg, Austria; 5 Institute for Physiology and Pathophysiology, Paracelsus Medical University, Salzburg, Austria; 6 Department of Internal Medicine I, Paracelsus Medical University / Salzburger Landeskliniken, Salzburg, Austria; Universidade Federal de Pernambuco, BRAZIL

## Abstract

Analyses of the Journal Impact Factor (JIF) have grown to be a major topic in scientometric literature. Despite widespread and justified critique concerning the JIF and its application, the size of a journal as a predictor for its longitudinal variability–or stability–on a long-term level has not yet comprehensively been analyzed. This study aims to provide robust evidence for an association between JIF variability and the size of journals, expressed by the number of published articles (citable items). For this purpose, the complete set of journals included in the Incite Journal Citation Reports (JCR) with an JIF in the 2017 JCR edition (n = 8750) were analyzed for the association between journal size and longitudinal JIF dynamics. Our results, based on n = 4792 journals with a complete JIF data set over the timespan of 12 annual JIF changes show that larger journals publishing more citable items experience smaller annual changes of the JIF than smaller journals, yet with this association being reversed for journals with a very large number of total cites. Consequently and in accordance with the genuine intention of the JIF to serve as a basis for decisions on journal subscriptions, evaluation of current changes of the JIF have to be accompanied by consideration of the journal’s size in order to be accurate and sensible.

## Introduction

The Journal Impact Factor (JIF) is published on an annual basis as part of the Incites Journal Citation Reports (JCR) by Clarivate Analytics and currently represents the most frequently used journal-based metrics (e.g. [1, 2]). Initially proposed by E. Garfield in 1955 [3] and probably earlier in 1927 by Gross P.L. [4], it was designed as a measure of journal quality in order to aid librarians to decide which (subscription-based) journals should be purchased for the institution’s library. While using the JIF to evaluate individual researchers or institutions is highly controversial (also pointed out by E. Garfield [5]), this approach is currently used at numerous institutions [6] and frequently criticized as misuse of the JIF, see e.g. [7].

There are several and general points of critique regarding the JIF such as the inappropriate use of an arithmetic mean (JIF) for an asymmetric distribution (citations per papers for each journal follow a highly skewed Pareto-like distribution, known as Bradford’s law in bibliometrics [8]), the potentially distortional effects of the denominator in the JIF equation (e.g. [9]), and the (arbitrary) two-years window the JIF calculation is based on (e.g. [2]). Additionally, even when correctly used as a measure of journal performance, the currently highly competitive field of academic publishing (“big business” [10]) has made some journal editors to develop several strategies for increasing their JIF–already described about ten years ago [11–14]. Besides such strategies and based on the original intention as a measure of journal performance, editorials are frequently published on the (usually positive) development of the own journal’s JIF (see PubMed using ‘impact factor [ti]’ and restrict article type to ‘editorials’], thus advertising to promote visibility and attractiveness for potential authors and purchasers.

Due to the nature of the JIF as an average (mean) which is highly sensitive towards outliers (e.g. highly-cited papers), the longitudinal trend of a journal’s JIF might be subject to statistical fluctuations, i.e. a random component in JIF changes. The size of this component expressed as the relative standard deviation (SD) has been estimated in the range between 7% and 34% [14, 15] for journals publishing about 60–70 papers per year. Besides relativizing pseudo-precision suggested by the JIF formula, this phenomenon has implications for comparing journals with respect to their JIF development over time: as demonstrated by Amin and Mabe for a n = 4000 sample of journals and a single JCR transition from 1997 to 1998, the mean changes in JIF are highly dependent on the journal’s size (articles published per year), i.e. more than ±40% and about ±18% annual JIF changes for journals publishing <35 and >150 articles per year, respectively. Similarly, a range of 9–18% relative SD for the trend in the years 2001–2004 has been shown by Ogden and Bartley for a set of ten journals in the field of occupational/environmental health/hygiene [14].

As both journals and publishers legitimately promote their product by referring to (positive) changes of the JIF over time and decision makers must be able to appraise such statements based on a reasonable and sensible basis, the current study aims to provide a comprehensive analysis of longitudinal JIF changes in relation to the size of the respective journals. For this purpose, the complete collection of journals indexed in the JCR has been analyzed for JIF changes and its association with journal size within a longer period, i.e. the JCR years 2005–2017. The data were analyzed by descriptive statistics, followed by a regression model taking into account the size and total cites of the journal. Based on this comprehensive and longitudinal analysis, the (negative) association between journal size and JIF variability as well as potential interactions between the size and total cites of the journal are demonstrated.

## Material and methods

### Data retrieval and representation

From the JCR database, a list of all SCIE journals (Science citation index expanded) including the journals’ JIF, total cites (TC), and citable items (CI) for each JCR year has been downloaded on the 9^th^ of July 2018 using an institutional subscription via https://jcr.clarivate.com. Journals were excluded from further analyses if no JIF was available for the last JCR’s edition (2017). Visualization of results were generated using OriginPro 2019b (OriginLab Corp., Northampton, MA, USA) and Corel Designer 2018 (Corel Corp., Ottawa, Canada). Statistical analyses were performed using R version 3.5.1 (R Core Team 2018) [16].

### Statistical methods

**Fig 1:**
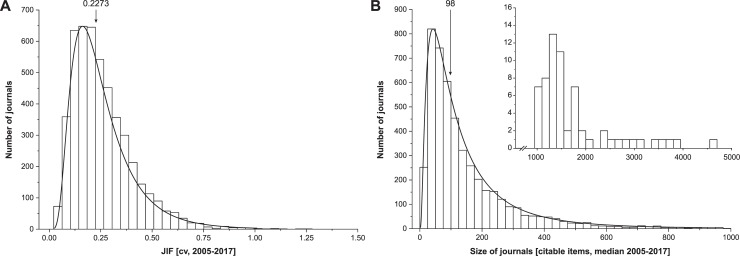
Distribution of the JIF variation coefficient (cv) and the size of journals (CI). Histograms show the relative frequency of journals according to their JIF’s variability (coefficient of variation (cv) of the journal impact factor) in panel (A) and according to their size (citable items), panel (B). In both histograms, the curve indicates a lognormal data fit and the arrow indicates the median. The insert in panel B shows the histogram for journals >1000 citable items. Journals with complete data sets for JCR and CI for the JCR years 2005–2017 are included (n = 4792).

For the primary analysis, the coefficient of variation (cv) was calculated for journals where JIF and CI data were available for all JCR years between 2005 and 2017. The cv is defined as the standard deviation divided by the arithmetic mean and, thus, quantifies the factor by which the standard deviation exceeds the mean (e.g., if the mean is equal to the standard deviation, the cv is equal to 1; if the mean is equal to, say, 2, but the standard deviation is 4, this yields a cv of 2). However, since for the journals analyzed, the distribution of cv turned out to be skewed (Fig 1A), the natural log of the cv was considered in all subsequent analyses. The cv is supposed to be more appropriate than taking the raw standard deviations with respect to comparing different journals, due to the normalization by the mean when calculating the former quantity. Anyway, we found that the distribution of the log standard deviations was very similar to the distribution of the log cv values. The same applies to taking the interquartile range and the interquartile range divided by the median, respectively. This altogether indicates that the results do not depend on the quantity that is used for quantifying JIF variability over time.

The size of a journal was assumed to be represented by the median number of CI throughout all JCR years between 2005 and 2017. Again, we took the natural logarithm of CI, in order to reduce the skewness of the data. Additionally, we also considered a categorized size variable, which was created by splitting the data at the quartiles based on the median CI.

In addition to basic descriptive analyses, we estimated the correlation between log(cv) and log(CI) by means of the Spearman correlation coefficient, because it is invariant under strictly monotone transformations (e.g., logarithmic transformations). The 95% confidence interval was calculated using the bootstrap percentile method. Moreover, we used a multiple linear regression model in order to explore the associations between the dependent variable log(cv) and the explanatory variables log(CI) as well as the logarithm of the median number of total cites (TC) in more detail. To investigate the robustness of the results further, we repeated all analyses described above, using the data from all journals (including those with incomplete JIF or CI sets in the JCR years 2005–2017) where calculation of log(cv), log(CI) and log(TC) was possible (n = 8750 journals).

## Results

### Descriptive analysis

Overall, data of 8996 journals were considered. Complete data sets for all JCR years between 2005 and 2017 was available for 4792 journals. Within the latter, the distributions of CV and size are highly asymmetric, with a median cv = 0.23 of the JIF (IQR = 0.15–0.33, range = 0.02–1.25) and a median size (CI) = 98 (IQR = 53–190, range = 3–4677), see Fig 1. Applying the log-transformation yields distributional shapes close to a normal distribution.

**Fig 2:**
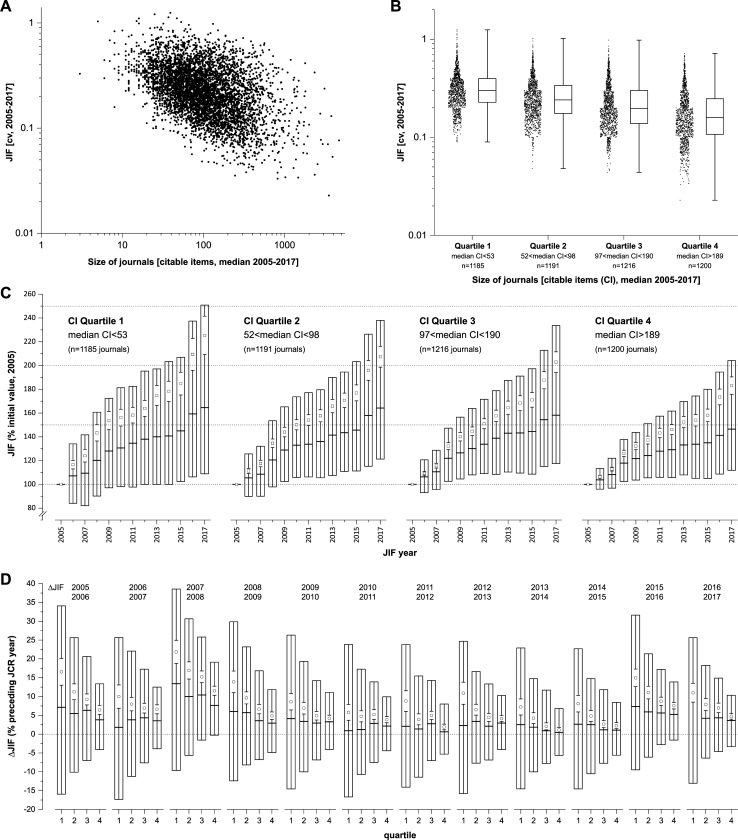
Association between JIF variability (coefficient of variation) and the size of journals (CI). (A) Double-logarithmic scatter plot for the JIF’s cv and size of journals (CI, citable items). (B) Box plots showing the 25^th^ and 75^th^ percentiles, median (horizontal line) and range (whiskers) for the four quartiles of journal size (q1-q4). (C) Longitudinal trend of the JIF related to the initial value (2005 JCR) for each journal. The box plots show the 25^th^ and 75^th^ percentiles, median (horizontal line), mean (open square) and 95% confidence interval (whiskers). (D) The relative changes in JIF (% preceding year) are shown for q1-q4 (box plots were designed as in (C)). In all graphs, journals with complete data sets for JCR and CI for the JCR years 2005–2017 are included (n = 4792).

Fig 2A and 2B indicate a negative linear association between log(CV) and log(size), although there is also a considerable amount of spread which might complicate a clear and unambiguous interpretation. Accordingly, a moderate negative correlation of -0.42 (95% CI [-0.44, -0.39]) was found. Fig 2C shows the longitudinal trend for journals with a complete data set for the JCR years 2005–2017 relative to the initial JCR year (2005): while in all four quantiles q1-q4), the mean and median JIFs show a clear increase during this period (up to 160% (median) and 220% (mean) related to the initial JCR year 2005), the variability of data (25^th^ and 75^th^ percentiles and 95% confidence interval) is considerably decreasing from (small) q1 journals (CI<53) to large q4 journals (CI>189). Based on the presentation used by Amin and Mabe [15], i.e. when the relative JIF changes (% of preceding JCR year) are plotted per quartile journal size (Fig 2D), again, the trend for smaller JIF changes in the case of large journals is obvious: roughly, for all annual changes of the JIF, the interquartile range for large journals in q4 (e.g. +10 to -3 JIF points for the JCR transition 2016–2017) is just about one third of that of small journals in q1 (e.g. +25 to -12 JIF points for the JCR transition 2016–2017). While all mean and median values of these data sets are above 0% ΔJIF thus indicating a consistent annual JIF increase, larger journals (q4) tend to show less pronounced JIF increases.

### Model on quantitative association of JIF changes and journal size

The results of a multiple linear regression model with log(cv) as dependent variable and log(CI), log(TC) and the interaction between log(CI) and log(TC) as explanatory variables are provided in Table 1.

**Table 1:** Multiple linear regression for n = 4792 journals with complete JCR data (2005–2017).

	Estimate (SE)	Test statistics	P value
Intercept	0.738 (0.158)	-	-
Log(CI)	-0.087 (0.035)	-2.461	0.0139
Log(TC)	-0.292 (0.021)	-14.116	2*10^−16^
Interaction	0.011 (0.004)	2.784	0.0054

Abbreviations: CI = median number of citable items, TC = total cites, Interaction = interaction between log(CI) and log(TC), Estimate = estimated regression coefficient, SE = standard error.

Before conducting the analyses, the adequacy of the model was assessed using residual plots, which revealed a decent fit. In line with the abovementioned correlations, the estimated coefficient for log(CI) was -0.087 (SE = 0.035), thus indicating a negative association between log(CI) and log(cv). Interestingly, however, for journals with a relatively large number of total cites, the association could even be positive, as indicated by the positive coefficient of the interaction term. For example, consider a journal with 100 citable items and 20,000 total cites. Then, according to the linear regression model, the average predicted cv would be 0.131 (95% prediction interval: 0.053–0.322). Now, if the number of citable items was increased to 600, the average predicted cv would be 0.137 (95% prediction interval: 0.056–0.338).

As a sensitivity analysis, calculation of the Spearman correlation and the regression coefficients was repeated using data from all journals where the cv, CI and TC could be determined (n = 8750). The correlation between the cv of the JIF and the CI was very similar to above (Spearman’s rho = -0.40, 95% CI [-0.42, -0.38]). Likewise, the results of the linear regression model are more or less the same, except the weaker interaction between log(CI) and log(TC), see S1 Table.

## Discussion

As a comprehensive extension and update of previous studies [14, 15], the current investigation including data for twelve annual JIF changes provides robust evidence for an association between JIF variability and the size of journals. In particular, larger journals publishing more citable items show smaller annual changes of the JIF when the latter has been expressed as the coefficient of variation, i.e. as a relative measure of JIF variability.

In their article, Amin and Mabe [15] explained JIF variation as a consequence of statistical effects relating to the number of items being averaged, i.e. the number of citable items or the size of the journal. The size of the JIF window (a 1-year citing and a 2-year cited period) additionally limits the size of statistical sample being considered by the JIF formula. For a sample of 4000 journals and the transition between 1997 and 1998 JIFs, the authors observed a ±40% JIF average change in JIF for small journals (<35 papers per year) while large journals with >150 articles per annum showed on average about only ±18% JIF. The current study also has used quartiles to define groups of journals according to their size (CI) and we found similar effects of these characteristics on the JIF variability: i) there is a negative correlation between the JIF’s coefficient of variation and the size of journals (Fig 2A), ii) the median coefficient of variation is nearly two times higher in small journals (cv = 0.30 for CI<53, q1) compared to large journals (cv = 0.16 for CI>190, q4; Fig 2B), iii) in the longitudinal trend (JIFs 2006–2017 related to 2005; Fig 2C), the JIF’s interquartile range as well as the confidence interval is considerably smaller in large versus small journals, and, iv) direct comparison of the relative annual ΔJIFs between journal quartiles indicate a consistently smaller variability for large versus small journals (Fig 2D). The results from multiple linear regression analyses were basically in line with those findings, although we have also found some indication that the effect might be altered by the number of total cites. Anyway, in addition to the aforementioned descriptive results, the regression model allows for predictions regarding the cv, given the journal size and the number of total cites, and hence may serve as a convenient tool to aid in decision making.

Interestingly, while Amin and Mabe described a symmetric distribution of mean changes in JIF (1997–1998) around zero, i.e. an equal proportion of journals increasing or decreasing their JIF [15], we observed a clear trend towards annually increasing JIFs (i.e. mean/median JIF changes >0%). While this effect is more pronounced for smaller journals, this observation is in accordance with several publications describing a general ‘inflation’ of the JIF over time [9, 17, 18].

Ogden and Bartley analyzed the JIF of the journal ‘Annals of Occupational Hygiene’ and nine other journals in that field (all with JIFs in 2001–2004 averaging within 0.58–1.94) and found a relative JIF standard deviation in the range of 9–18% within this period [14]. While these numbers are smaller than suggested by Amin and Mabe [15], both articles conclude that observed changes in a journal’s JIF might well be attributable to simple statistical variations, i.e. are in the range of random fluctuation due to limited sampling sizes [14, 15]. For example, based on a theoretical model assuming unbiased/random sampling, Amin and Mabe stated that a journal publishing 140 articles need to experience a ±22% JIF change to be significant, i.e. exceeding the range of random JIF fluctuation. In other words, “An impact factor of 1.50 for a journal publishing 140 articles is not significantly different to another journal of the same size with an impact factor of 1.24” [15]. The authors further extrapolate that regarding the JCR journal ranking (as a rule of thumb), journals with JIFs differing “<25% belong together in the same rank” and that it would be inappropriate to set certain JIF-based thresholds for publishing, e.g. to “penalize authors for publishing in journals with JIF less than a fixed value, say, 2.0, given that for the average-sized journal, this value could vary between 1.5 and 2.25, without being significant” [15]. In line with this notion, Science’s former Editor-in-Chief, Bruce Alberts, regarded it manifestations of the misuse of the JIF [19] or the nonsense JIF ‘mania’ if researchers prepare their curriculum vitae including publications annotated with the respective journal’s JIF including three significant decimal places, or if publications in journals with JIFs < 5 are officially of zero value in some countries [20].

“A ripple of excitement passes through the scientific community” [21] each year, when the new edition of the JCR is published and “the release of these results triggers elation or gloom in editorial offices around the world” [21]. “Publishers and editors celebrate any increase, whereas a decrease can send them into a huddle to figure out ways to boost their ranking” [19] and “editors break open bottles of champagne if their impact factor rises by a tenth of a decimal point or burst into tears if it falls” [22]. While polemic in nature, these quotations adopted from published comments on the matter illustrate the journals’ interest in high or at least increasing JIF-based metrics–in accordance with the genuine intention of the JIF [3–5], i.e. to aid librarians in deciding which journals their institution should subscribe. Accordingly, Clarivate Analytics lists as the first item in the target audience of the JCR the “Librarians–to support selection or removal of journals from their collections, and to understand how their researchers are contributing to that journal” [23]. In light of the data shown in the present study, it seems worth mentioning that in the publisher’s blog advertising for the current release of the 2018 JCR, the company explicitly mentions the journal ‘CA-A Cancer Journal for Clinicians’ as that experiencing the highest increase in its JIF (from 187.040 to 244.585 for JRC 2016 to 2017) [24]. As this journal falls into Q1 of the present study, i.e. publishing <53 citable items per year (29 articles in 2017), a high variability of its JIF is not surprising–in fact, this journal has experienced annual changes of its JIF in the magnitude of +50 JIF points three times in the past decade (between JCRs 2011–12, 2015–16 and 2016–17). Though such absolute numerical increases of a JIF are quite impressive–other journals probably would dream of such a number as their JIF, not as its change–, considering the journal’s size relativizes these figures.

In this context, the data of this study highlight the need for careful evaluation of changes in the JIF because their extent is strongly dependent on the size of the journal, i.e. small journals might experience sparkling annual increases in the JIF, but much of that might be due to a small sample size. In other words, when deciding on whether journals are worth being subscribed for e.g. an institution’s library, evaluation of the current changes in the JIF have to be accompanied by consideration of the journal’s size in order to be sensible. Clearly, other criteria might influence the choice of journal subscriptions at an academic library including costs, demand (faculty interest), subject and collection balance, access restrictions, back files etc. Furthermore, journals are mostly not subscribed as individual publications but rather as (e-journal) packages provided by publishers. Therefore, the JIF does not represent the only determining aspect for librarians’ decisions and counter statistics offered by the various publishers’ platforms provide additional (ex post) information for the librarians on faculty usage of the subscribed journals. With the increasing importance of open access publishing models (probably in future representing the mainstream model of scholarly publishing [25]), such decisions will, additionally, be less relevant due to a transition from subscription-based to author-charge-based financing of journals and publishers. Since, however, both the prestige of individual articles and researchers’ or institutions’ performance is frequently (and abusively) measured using the JIF (e.g. [7, 15, 19, 26, 27]), also the choice of a target journal for manuscript submissions–if influenced by JIF (changes)–should consider the longitudinal stability of the JIF and thus indirectly the size of the journal(s) under consideration. For this purpose, the current study demonstrates journal size as a valid and easily retrievable surrogate parameter for a journal’s JIF variability.

## Conclusions

Journal size in terms of citable items is a strong predictor of the journal’s longitudinal JIF stability and needs to be considered when current changes of a journal’s JIF are evaluated. This applies e.g. for librarians’ decisions on future journal subscriptions or for authors, provided that JIFs constitute a part of the criteria employed in the decision on where to submit a research manuscript.

## Supporting information

S1 Table Multiple linear regression for n = 8750 journals including journals with complete (n = 4792) and partly incomplete JCR data (2005–2017). (DOCX)
