# Supplementary material for: Size matters! Association between journal size and longitudinal variability of the Journal Impact Factor
Source: PLoS One. 2019 Nov 22;14(11):e0225360. doi: 10.1371/journal.pone.0225360 (PMC6874322; doi:10.1371/journal.pone.0225360)
Supplement: S1 Table — (DOCX) [file pone.0225360.s001.docx]

**Supporting Information**

**S1 Table. Multiple linear regression for n = 8750 journals including journals with complete (n=4792) and partly incomplete JCR data (2005-2017).**

|  | Estimate (SE) | Test statistics | P value |
| --- | --- | --- | --- |
| Intercept | 0.176 (0.102) | - | - |
| Log(CI) | -0.078 (0.024) | -3.224 | 0.0013 |
| Log(TC) | -0.195 (0.014) | -13.522 | 2*10^-16^ |
| Interaction | 0.003 (0.003) | 1.154 | 0.2483 |

Abbreviations: CI = median number of citable items, TC = total cites, Interaction = interaction between log(CI) and log(TC), Estimate = estimated regression coefficient, SE = standard error.
